# Stem Diameter (and Not Length) Limits Twig Leaf Biomass

**DOI:** 10.3389/fpls.2019.00185

**Published:** 2019-02-21

**Authors:** Jun Sun, Mantang Wang, Min Lyu, Karl J. Niklas, Quanlin Zhong, Man Li, Dongliang Cheng

**Affiliations:** ^1^Fujian Provincial Key Laboratory of Plant Ecophysiology, Fujian Normal University, Fuzhou, China; ^2^School of City and Civil Engineering, Zaozhuang University, Zaozhuang, China; ^3^Key Laboratory of Humid Subtropical Eco-Geographical Process, Ministry of Education, Fuzhou, China; ^4^Plant Biology Section, School of Integrative Plant Biology, Cornell University, Ithaca, NY, United States

**Keywords:** allometry, annual growth, biomass allocation, elevation gradient, forest types, isometry, stem architecture

## Abstract

The relationship between leaf and stem biomass as well as the relationship between leaf biomass and stem length and diameter are important to our understanding of a broad range of important plant scaling relationship because of their relationship to photosynthesis and thus growth. To understand how twig architecture (i.e., current year leaves, and stem diameter and length) affects stem diameter and length, and leaf number and biomass, we examined the twigs of 64 woody species collected from three forest types along an elevational gradient in the Wuyi Mountains, Jiangxi Province, China. We also compared the scaling relationships we observed with biomass allocation patterns reported at the whole tree level. Our results revealed isometric relationship between leaf and stem biomass on twigs despite differences in forest communities and despite changes in environmental factors along an elevational gradient. Across the 64 species, from twigs to individual trees, leaf biomass scaled approximately as the 2.0-power of stem diameter (but not for stem length or leaf number). These results help to identify a general rule that operates at two different levels of biological organization (twigs and whole trees). The scaling relationship between leaf biomass and stem diameter in twigs is insensitive to differences in species composition, elevation, or forest type. We speculate that this rule emerges because stem diameter serves as a proxy for the amount of resources supplied per unit cross section to developing leaves and for the flow of photosynthates from mature leaves to the rest of the plant body.

## Introduction

Regardless of their form, size, or longevity, the leaves on current-year shoots (i.e., twigs) provide the photosynthetic machinery that drives annual growth, whereas the stems of twigs sustain the static and dynamic mechanical forces leaves experience by gravity and wind (i.e., self-loading and wind induced drag forces, respectively) (Niklas, 1992a). The hydraulic architecture of mature twigs also provides for the efflux of photosynthates and influx of water and nutrients. Consequently, quantifying the scaling relationships between leaf and stem biomass, and the relationship between stem diameter and length is critical to our understanding of a broad spectrum of important ecological phenomena (Westoby and Wright, 2003; Niklas and Spatz, 2004; Sun et al., 2006; Olson et al., 2009; Xiang et al., 2009a).

Generally, the allocation of biomass to leaves, stems, and roots can be described using a scaling function (Enquist and Niklas, 2002), which takes the general mathematical formula *M_a_* = β*M_b_*^α^, where *M_a_* and *M_b_* are the biomass of different organs, β is the normalization constant, and α is the scaling exponent. Prior investigations using this formula have focused not only at the whole-plant level (Enquist and Niklas, 2002; Cheng and Niklas, 2007; Chave et al., 2014; Paul et al., 2016), but also at the level of individual twigs (e.g., Westoby and Wright, 2003; Sun et al., 2006; Xiang et al., 2009a; Yang et al., 2015). For example, an isometric relationship is reported for the scaling of leaf and stem growth (i.e., *G_L_* ∝*G_S_*) (Niklas and Enquist, 2002b). Therefore, it is not unreasonable to speculate that total leaf biomass might scale isometrically with respect to total stem biomass at the twig level. Indeed, some empirical results indicate that leaf biomass scales nearly isometrically with stem biomass at the level of individual twigs (Sun et al., 2006; Xiang et al., 2009a). However, other interspecific studies indicate that the smaller twigs might have a larger ratio of leaf to stem biomass (Yagi, 2000; Suzuki, 2003; Pickup et al., 2005; Wright et al., 2006). Indeed, when viewed collectively, there is considerable ambiguity about how the key functional traits of twigs (e.g., leaf and stem biomass, and stem diameter and length) actually scale with respect to one another, especially for different plant communities growing along environmental gradients, such as an elevational gradient.

In order to clarify the scaling relationships of twigs along the elevational gradient, we developed a mathematical model for the scaling of critical twig functional traits and we tested the model using data gathered from 915 twigs from 64 woody species in three different forest-types (i.e., deciduous, mixed forest, and evergreen forest) along an elevational gradient in the Wuyi Mountains. The model and the data were used to determine (1) twig biomass allocation patterns, and (2) how leaf biomass scales with respect to stem diameter and length at the twig level along a steep (2158 m) elevational gradient. Using published data, we also compared twig scaling relationships to those at the whole-tree level to provide a comprehensive view of plant biomass allocation patterns at different levels of biological organization (i.e., primary and secondary growth as well as two branching levels within tree-sized plants).

## Materials and Methods

### The Model

Our model rests on the assumpt that a positive scaling relationship exists between stem diameter and total leaf biomass because (1) the transport of water and nutrients to transpiring leaves is likely to scale positively with stem cross sectional conductive area (and thus diameter) (Shinozaki et al., 1964; Niklas and Spatz, 2004) and because (2) the mechanical capacity of stems to resist bending and torsion is positively correlated with the second moment of area (and thus diameter) (Niklas and Spatz, 2010). The theoretical relationship between stem diameter and length at the twig level is less clear. However, it is reasonable to surmise that, for any stem biomass, a negative correlation exists between stem diameter and length, provided that the bulk density of stem tissues is invariant (Niklas and Spatz, 2010). Accordingly, stem diameter and length should negatively correlate with wood density. Turning to the whole plant level, prior research indicates that plant height (*H*) scales approximately isometrically with respect to basal stem diameter (*D*) across seedlings and non-woody or very small woody species (i.e., *H* ∝*D*), but as the 2/3 power of *D* across the larger trees (i.e., *H* ∝*D*^2/3^) (Niklas and Spatz, 2004; see, however, Zhang et al., 2016). Although these relationships do not directly bear on the scaling of stem diameter with respect to stem length on twigs, a similar tactic can be taken by relating the scaling of basal stem diameter to plant height. Following the same logic as before, it is reasonable to suppose that twig length (*L*) might scale isometrically with respect to twig diameter (*D*) (i.e., *L* ∝*D*). Assuming that individual stems are more or less cylindrical in geometry, it follows that stem biomass (*M_S_*) will be proportional to the product of the square of diameter and stem length (i.e., *M_S_* ∝*D^2^L*). Assuming an invariant bulk tissue density, twig stem mass will scale as the cube of either diameter or length (i.e., *M_S_* ∝*D*^3^ ∝*L*^3^). However, Xiang and Liu (2009b) report that *L* may be uncorrelated with stem diameter (*D*) due to phylogenetic reasons at twigs level. Therefore, whether leaf biomass scale as the cube of stem diameter at the twig level remains unclear.

Another variable of interest is leaf number because the total leaf biomass of twigs is the product of leaf number and the biomass of individual leaves. Assuming that stems can sustain a critical maximum leaf biomass (by virtue of hydraulic or mechanical limitations) (Westoby et al., 2002), the biomass of individual leaves must scale inversely with respect to total leaf number (Kleiman and Aarssen, 2007). For example, studies of leafing intensity (leaf number per unit stem volume) indicate that average leaf biomass increases with decreasing leaf number per stem volume (Kleiman and Aarssen, 2007; Milla, 2009; Huang et al., 2015). If this negative isometric relationship holds true across species, total leaf biomass should be independent of leaf number. However, a positive relationship between leaf number and total (and individual) leaf biomass has been reported at times (Smith et al., 2017). Therefore, it is still unclear how twig traits limit the total leaf biomass production across different forest communities along elevational gradients. Consequently, an empirical approach was adopted.

### Study Site Description

The study site is located in National Natural Reserve of Wuyi Mountains (27°48.11′–28°00.35′ N, 117°39.30′–117°55.47′ E). The reserve is located in the humid warm subtropics in the southeast of China and has a mean annual precipitation of 2583 mm and a mean annual temperature of 14.2°C. The forests growing on Huanggang were selected for study because (1) this mountain is the highest peak in the reserve at 2158 m with a mean above sea level (m a.s.l) of 1200 m and thus provided considerable climatic variation along its elevational gradients, and (2) because the vertical zonation of vegetation types along these gradients ranges from evergreen forests in the lower elevations to mountainous steppe in the higher elevations. The major soil types of Huanggang are classified as mountain yellow-red soil (400–600 m a.s.l), mountain yellow soil (600–1300 m a.s.l.), mountain dark yellow-brown soil (1300–1900 m a.s.l.) and mountain meadow soil (above 1900 m a.s.l.) (Li et al., 2017).

### Twig Sampling

Three forest communities were selected along an elevational gradient: (1) an evergreen forest (EF) located at 1319 m a.s.l, (2) a mixed forest (MF) located at 1697 m a.s.l, and (3) a deciduous forest (DF) located at 1818 m a.s.l. Three 20 m × 20 m plots were randomly established in each forests. Forest stand density, stem diameter at breast height (DBH), plant height (H) and soil nutrient content were measured for woody species within each plot. Sample information shown in Table 1.

**Table 1:** Ecological and morphometric traits of the three forest types examined in this study.

Forests	Altitude (m)	*n*	Density (trees/hm^2^)	Height (m)	Mean DBH (cm)	Soil carbon content (mg/g)	Soil nitrogen content (mg/g)	Soil phosphorus content (mg/g)
EF	1319	32	3033 ± 200a	7.87 ± 0.07b	13.77 ± 1.46b	68.88 ± 0.59a	4.84 ± 0.04a	0.46 ± 0.01b
MF	1697	20	1133 ± 164b	10.56 ± 0.21a	21.39 ± 0.8a	78.71 ± 4.36a	5.25 ± 0.27a	0.38 ± 0.02c
DF	1818	23	2725 ± 164a	6.94 ± 0.24b	11.47 ± 0.67b	75.16 ± 5.23a	6.05 ± 0.22a	0.65 ± 0.01a


*EF, evergreen forest; DF, deciduous forest; MF, mixed forest. Using one-way ANOVA, the values of morphometric functional traits and soil stoichiometry within columns not sharing a common letter are significantly different (*post hoc* LSD test, α = 0.05).*

As noted in the Introduction, a twig is defined as a first-year shoot, consisting of a stem and attached leaves. In August, 2016, three to five individuals of each species were randomly selected, and 5 twigs were taken from the perimeter of the crown per individual plant. For species with less than three individuals in a plot, five twigs from each individual were harvested. The total number of species was 64, spanning 27 families and 45 genera among the three forest types. Specifically, 32, 20, and 23 species (including overlapping species) were collected in the evergreen, mixed, and deciduous forests, respectively. The dominant species in the EF were *Rhododendron simiarum*, *Schima superba*, *Cyclobalanopsis glauca*, *Rhododendron ovatum*, and *Symplocos sumuntia*. The dominant species in the MF *Symplocos sumuntia* and *Cyclobalanopsis multiervis*, *Tsuga chinensis*, *Taxus chinensis*, *Acer elegantulum*, and *Illicium minwanense*. The dominant species in the DF *Clethra barbinervis*, *Photinia beauverdiana*, *Acer nikoense*, and *Fraxinus chinensis*. All of the leaves on each twig were removed and each leaf was scanned to measure its area using the software Image J. Stem diameter at the top, middle, and bottom of each twig was measured and used to calculate a mean diameter. Twig length was measured using a vernier caliper, with an accuracy of 0.1 mm. Stem volume was calculated as the square of stem diameter times stem length, assuming that individual stems were more or less cylindrical in geometry. All leaves and stems attached on twigs were subsequently brought to the laboratory and oven-dried at 75°C to determine total leaf biomass (*M_L_*), total stem biomass (*M_S_*), and total biomass (the sum of leaf and stem biomass, *M_T_*). Wood density (ρ) was calculated as stem biomass / stem volume.

The scaling relationships governing twig biomass allocation patterns were compared with those at the whole tree level. A total of 1123 records for total leaf, total stem, and total plant size (*M_L_*, *M_S_*, and *M_T_*, respectively) and 548 records for plant height and DBH complied by Cannell (1982); Enquist and Niklas (2002), and Niklas and Enquist (2002a,b) were analyzed. The data included measurement taken on eudicot, monocot, and conifer species, and from seedlings and reproductively mature tree species.

### Data Analysis

For each species, the mean values for all twig traits were calculated and used. All of the data were log_10_ transformed to fit a normal distribution before analysis. The relationships between all twig functional traits were best fit by the mathematical equation log (*y*) = log (β) + αlog (*x*), where β is the normalization constant and α is the scaling exponent. Model Type II regression was used to determine the numerical values of β and α using the (Standardized) Major Axis Estimation package ‘smatr’ version 3.4-3 in R software (R Core Team, 2012; Warton et al., 2012). The data from species showing no statistically significant differences in the numerical values of the two regression parameters were pooled to determine a common scaling exponent using the standardized major axis package in R (Warton et al., 2006, 2012). The significance level for testing slope heterogeneity was *P* < 0.05 (e.g., slope heterogeneity was rejected when *P* > 0.05). Further, in order to determine whether the correlation between different functional traits varied with evolutionary divergence, the phylogenetic signals of twig functional traits in the three forests were examined using Phylogenetically Independent Contrast Analysis (PIC), which was calculated using the “*pic*” function in the “ape” package in R 3.4.3 software (Paradis et al., 2004). The *K*-value method proposed by Blomberg et al. (2003) measures the intensity of phylogenetic signals of continuous functional traits, which was calculated using the “*phylosignal*” function in the “picante” package in R 3.4.3 software (Kembel et al., 2010). *K* > 1 indicates that functional traits exhibit a stronger phylogenetic signal; *K* < 1 indicates that the functional traits exhibit weak phylogenetic signals (Blomberg et al., 2003).

A structural equation model was used to create an empirical model for predicting how traits influence total leaf biomass at the twig level. The model run through SPSS AMOS 22.0 (SPSS. Inc., Chicago, IL, United States). Specifically, we constructed an initial model for expected causal relationships between total leaf biomass and twig trait variables based on prior theoretical knowledge (Figure 3A). Because the initial model did not provide a good fit to the data, the SEM was simplified and evaluated using maximum likelihood chi-squared tests (Grace et al., 2007). The CMIN/DF (the ratio of Chi-Square test value and degrees of freedom), GFI (Adjusted Goodness-of-fit Data), NFI (Normed Fit Index) were used to determine whether the fit between the simplified model and data was adequate.

## Results

### Biomass Allocation Patterns

**FIGURE 1:**
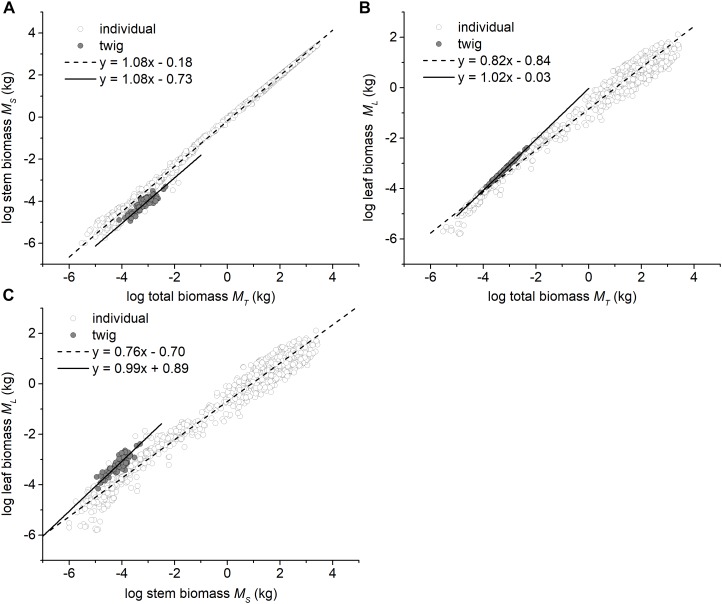
Bivariate plots among the leaf, stem, and total biomass at the twig level and the individual plant level. **(A)** The relationship between stem and total biomass, **(B)** The relationship between leaf and total biomass, and **(C)** The relationship between leaf and stem biomass. The data of individual plants were taken from Cannell (1982), Enquist and Niklas (2002), and Niklas and Enquist (2002a,b).

The three traits (i.e., *M_L_*,*M_S_*, and *M_T_*) scaled nearly isometrically with respect to one another (i.e., α ≈ 1.0) across the three different forest communities along the elevational gradient (Table 2). Also, there was no statistically significant difference in the numerical values of the scaling exponents and normalization constants within each of the three different forest communities. Specifically, the common slopes were 0.99 (95% CI = 0.89 – 1.11, *P* = 0.13) for *M_S_* vs. *M_T_*, 1.01 (95% CI = 0.995 – 1.02, *P* = 0.19) for *M_L_* vs. *M_T_*, and 1.03 (95% CI = 0.91 – 1.15, *P* = 0.17) for *M_L_* vs. *M_S_* (Supplementary Figures 1A,B). Across the three forest communities, the scaling exponents of *M_S_* vs. *M_T_*,*M_L_* vs. *M_T_*, and *M_L_* vs. *M_S_* were 1.02, 1.01, and 0.99, respectively (Table 2), each of which was statistically indistinguishable from 1.0 (all *P*_1.0_ > 0.05) (Table 2). The scaling of stem biomass vs. total twig biomass across the three different forest communities was consistent with our model, i.e., the scaling had a common slope of 1.08 (95% CI = 1.07 – 1.08, *P* = 0.24) (Figure 1A).

**Table 2:** Summary of regression parameters (slopes and *y*-intercepts, α and log β, respectively) for relationships between leaf and stem biomass, and stem biomass (leaf biomass) vs. stem diameter (and length) in three forests.

	Forest type	N	α (95%CI)	log β (95%CI)	*r*^2^	*P*
*M_S_* vs. *M_T_*	EF	32	0.96 (0.77, 1.20)	–1.10 (–1.75, –0.46)	0.65	<0.001
	DF	23	1.17 (0.97, 1.43)	–0.37 (–1.11, 0.36)	0.81	<0.001
	MF	20	0.92 (0.80, 1.07)	–1.27 (–1.72, –0.83)	0.91	<0.001
	ALL	75	1.02 (0.92, 1.12)	–0.92 (–1.24, –0.60)	0.82	<0.001
*M_L_* vs. *M_T_*	EF	32	1.03 (1.01, 1.06)	0.05 (–003, 0.13)	0.99	<0.001
	DF	23	1.00 (0.96, 1.03)	–0.06 (–0.18, 0.05)	0.99	<0.001
	MF	20	1.01 (0.99, 1.02)	–0.02 (–0.06, 0.03)	0.99	<0.001
	ALL	75	1.01 (0.99, 1.02)	–0.03 (–0.07, 0.02)	0.99	<0.001
*M_L_* vs. *M_S_*	EF	32	1.07 (0.85, 1.36)	1.23 (0.19, 2.27)	0.59	<0.001
	DF	23	0.85 (0.68, 1.07)	0.25 (–0.54, 1.05)	0.75	<0.001
	MF	20	1.09 (0.93, 1.28)	1.37 (0.63, 2.11)	0.90	<0.001
	ALL	75	0.99 (0.89, 1.11)	0.89 (0.44,1.34)	0.78	<0.001
*M_S_* vs. *D*	EF	32	1.87 (1.40, 2.50)	1.44 (1.26, 1.62)	0.38	<0.001
	DF	23	2.13 (1.50, 3.03)	1.24 (0.97, 1.52)	0.38	<0.001
	MF	20	1.77 (1.35, 2.32)	1.25 (1.07, 1.42)	0.69	<0.001
	ALL	75	2.01 (1.70, 2.38)	1.34 (0.43, 2.26)	0.49	<0.001
*M_S_* vs. *L*	EF	32	1.22 (0.89, 1.67)	–0.18 (–0.89, –0.52)	0.26	<0.001
	DF	23	1.38 (0.98, 1.94)	–0.50 (–1.34, –0.34)	0.42	<0.001
	MF	20	1.92 (1.22, 3.02)	–1.25 (–2.65, 0.14)	0.11	<0.001
	ALL	75	1.44 (1.19, 1.74)	–2.24 (–2.61, –1.88)	0.32	<0.001
*M_L_* vs. *D*	EF	32	2.03 (1.68, 2.45)	2.39 (1.35, 3.44)	0.73	<0.001
	DF	23	1.81 (1.36, 2.42)	1.65 (0.22, 3.09)	0.61	<0.001
	MF	20	1.93 (1.55, 2.41)	1.97 (0.79, 3.15)	0.80	<0.001
	ALL	75	2.00 (1.75, 2.27)	2.22 (1.52, 2.92)	0.69	<0.001
*M_L_* vs. *L*	EF	32	–1.31(–1.89, –0.91)	5.26 (4.38, 6.14)	0.001	0.94
	DF	23	1.20 (0.77, 1.78)	0.73 (–0.16, 1.62)	0.11	0.14
	MF	20	2.10 (1.31, 3.38)	–0.55 (–2.17, 1.06)	0.01	0.64
	ALL	75	1.43 (1.14, 1.79)	–1.34 (–1.77, –0.90)	0.06	0.038
*M_L_* vs. *N_L_*	EF	32	1.45 (1.02, 2.07)	–4.11 (–4.49, –3.72)	0.04	0.40
	DF	23	1.68 (1.08, 2.61)	–4.46 (–5.05, –3.87)	0.04	0.27
	MF	20	–1.41 (–2.24, –0.89)	3.61 (3.08, 4.14)	0.06	0.31
	ALL	75	–1.54 (–1.94, –1.22)	–2.11 (–2.39, –1.84)	0.001	0.89


*M_T_, twig mass; M_L_, total leaf biomass; M_S_, stem biomass; D, stem diameter; L, stem length; and N_L_, leaf number.*

In contrast, at the whole tree level, the scaling exponents of *M_S_* vs. *M_T_*, *M_L_* vs. *M_T_*, and *M_L_* vs. *M_S_* significantly differed from 1.0 (i.e., α = 1.08, 0.82, and 0.76, all *P*_1.0_*<* 0.05, respectively; Figure 1A–C).

### The Scaling of Stem Architecture With Leaf Number, and Stem and Leaf Biomass

The scaling exponents of *M_S_* vs. *D* were statistically indistinguishable among the three forest communities (see Table 2 and Supplementary Figure 1C), with a common slope of α = 1.88 (95% CI = 1.59 – 2.24, *P* = 0.69). However, the numerical values of normalization constants for *M_S_* vs. *D* varied significantly, ranging from 0.87 for the mixed forest type to 1.09 for the evergreen forest type (Supplementary Figure 1C). Similarly, the scaling exponents of *M_S_* vs. *L* were indistinguishable among the three forests, with a common slope of α = 1.41 (95%CI = 1.14 – 1.73, *P =* 0.26). Additionally, the normalization constants of *M_S_* vs. *L* showed no significant difference among the three forests (i.e., β = -2.32, 95%CI = -2.66 – -1.97, *P* > 0.05) (see Table 2 and Supplementary Figure 1D).

**FIGURE 2:**
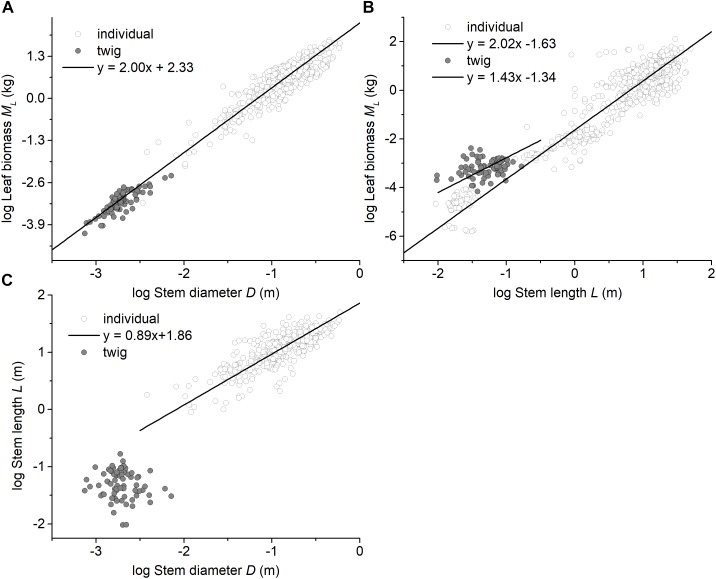
Bivariate plots of leaf biomass with respect to stem architecture at the twig level and at the individual level. **(A)** the relationship between leaf biomass and stem diameter, **(B)** the relationship between leaf biomass and stem height (length), and **(C)** the relationship between stem diameter and stem height (length). The data for individual plants were taken from Cannell (1982), Enquist and Niklas (2002), and Niklas and Enquist (2002a,b).

Among the three forest communities, the scaling exponents of *M_S_* vs. *D* were not statistically significantly different from 2.0 and had a common slope of α = 1.94 (95%CI = 1.72 – 2.20, *P =* 0.82) (see Table 2 and Supplementary Figure 2). However, the normalization constant for the data from the evergreen forest was significantly higher than that of other two forests (i.e., β = 2.17, 2.00, and 2.00 for Evergreen forest, Mixed forest, and Deciduous forest, respectively) (Supplementary Figure 2). Perhaps more important, the scaling exponent of *M_L_* vs. *D* across the three forests (i.e., α = 1.94) was statistically indistinguishable from that of the individual tree level (i.e., *M_L_* vs. *D*, α = 1.99), with a common slope of α = 2.00 (95%CI = 1.93 – 2.07, *P =* 0.98) (Figure 2A). In contrast, *M_L_* showed no statistically significant relationship with *N_L_* at the twig level within each or across all of the three forests (see Table 2 and Supplementary Figure 3). Although *M_L_* was weakly correlated with *L* (*P* = 0.038) across the three forests, no significant correlation was observed within each of the three forests (see Table 2). At the level of individual plants, both stem diameter and height were significantly correlated with total leaf biomass (all *P* < 0.001, Figure 2A,B).

Within each and across all of the three forest communities, no significant scaling relationship was observed for leaf number (*N_L_*) vs. stem diameter, or *N_L_* vs. stem volume (Table 3). However, *N_L_* scaled significantly with respect to *L* across the three forests, although little variation of *N_L_* was explained by *L* (Table 3). Although we found the leaf number had a phylogenetic signal, it had no discernable effect on the leaf number vs. total leaf mass relationship or other stem architecture traits (see Supplementary Table S1 and Supplementary Figure 4).

**Table 3:** Summary of regression parameters (slopes and *y*-intercepts, α and log β, respectively) for relationships between leaf number (*N_L_*) with respect to stem diameter (*D*), length (*L*), and volume (*V*) for all twigs in three forest.

	Forest type	*n*	α (95%CI)	log β (95%CI)	*r*^2^	*P*
*N_L_* vs. *L*	EF	32	–0.91 (–1.31, –0.63)	–0.41 (–0.83, 0.01)	<0.001	0.9
	DF	23	0.70 (0.46, 1.07)	1.62 (1.22, 2.02)	0.08	0.19
	MF	20	1.49 (0.97, 2.28)	2.86 (1.89, 3.83)	0.22	0.04
	ALL	75	0.93 (0.74, 1.16)	1.91 (1.63,2.19)	0.07	0.03
*N_L_* vs. *D*	EF	32	1.39 (0.97, 2.01)	4.47 (3.07, 5.88)	0.005	0.69
	DF	23	1.09 (0.70, 1.68)	3.65 (2.33, 4.97)	0.02	0.51
	MF	20	–1.37 (–2.15, –0.87)	–3.09 (–4.85, –1.33)	0.12	0.14
	ALL	75	–1.29 (–1.63, –1.03)	–2.81 (–3.63, –1.99)	0.01	0.35
*N_L_* vs. *V*	EF	32	0.64 (0.44, 0.91)	3.07 (2.18, 3.96)	<0.001	0.90
	DF	23	0.46 (0.30, 0.70)	2.46 (1.70, 3.21)	0.09	0.16
	MF	20	–0.69 (–1.10, –0.43)	–2.12 (–3.50, –0.73)	0.014	0.61
	ALL	75	0.57 (0.45,0.71)	2.86 (2.35, 3.37)	0.003	0.63


*EF, evergreen forest; DF, deciduous forest; and MF, mixed forest.*

**FIGURE 3:**
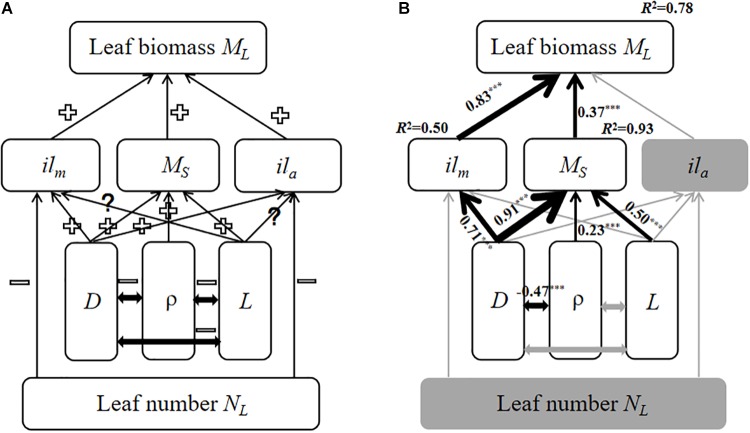
The models of the relationship among twig trait variables based on SEM. The gray solid arrows represent statistically non-significant correlations; the black solid arrows denote significant correlations in the final model. The thickness of the solid arrows indicates the magnitude of the standardized SEM coefficients, with its level of statistical significance (^∗∗∗^*p* < 0.001). **(A)** the initial model; **(B)** the final model. “+” indicates a positive relationship, “–” indicates a negative relationship. “?” indicates a unclear relationship. *il_m_*, Individual leaf biomass; *M_L_*, total leaf biomass; *M_S_*, stem biomass; *il_a_*, individual leaf area; *D*, stem diameter; ρ, wood density; and *L*, stem length.

The final structural equation model (SEM) predicting total leaf biomass at the twig level provided an adequate fit to our data, i.e., CMIN/DF = 2.2, GFI = 0.94, and NFI = 0.96. Increasing bulk stem tissue density was negatively correlated with stem diameter, but leaf number had no effect on individual leaf biomass nor individual leaf area. Increasing stem biomass and individual leaf biomass increased with total leaf biomass at the twig level (Figure 3B). More importantly, although stem length did influence stem biomass (Figure 3B), increasing stem diameter resulted in increasing stem mass and individual leaf mass (Figure 3B).

## Discussion

Although other studies of the relationships between leaf and stem biomass at the twig level have been published (Westoby and Wright, 2003; Pickup et al., 2005; Sun et al., 2006; Xiang et al., 2009a; Li et al., 2017), this study is the first to quantify the relationship and compare it among very different forest types along the elevational gradient. It is also one the few studies comparing the leaf and stem biomass relationship at the twig level with the individual-tree level. Thus, our analyses provide a novel opportunity to draw comparisons between two different but important levels of biological organization and to understand the mechanism(s) underlying leaf biomass allocation patterns.

### Biomass Allocation at Twig and Individual Level

At the twig level, our analysis shows that there are invariant and isometric scaling relationships among *M_S_*, *M_L_*, and *M_T_* (see Table 2 and Supplementary Figure 1) for three different forest types. This finding is consistent with some previous studies reporting isometric scaling exponents governing the relationships among these variables at twigs level (Sun et al., 2006; Xiang et al., 2009a). Further, the seemingly invariant isometric scaling relationship observed in our study supports previous reports that the total annual growth rates of leaves and stems at the whole plant level scales isometrically (Niklas and Enquist, 2002b). In contrast, our results are in apparent contradiction with other reports that large-leaved or thick-twigged species allocate more biomass to leaves (Pickup et al., 2005; Wright et al., 2006). One possible explanation for this inconsistency is the biomechanical trade-offs among a stem’s tissue bulk density, the geometric contribution to bending made by a stem’s cross-sectional area (called the second moment of area), and the effect of leaf mass (and area) on stem flexure (resulting from self-loading and wind-induced drag forces). The ability of a stem to resist bending of twisting can be increased by either increasing the material properties of its tissues (which, on average, increases with density), or by increasing its second moment of area by increasing its girth. Across species comparisons show that stems composed of weaker materials (those with lower material stiffness) compensate by having greater girth (Niklas, 1992b). Indeed, our results reveal that stem bulk tissue density is negatively correlated with stem diameter (Figure 3B).

Perhaps more important, out data show that stem biomass scales as the 1.08 power with respect to total shoot biomass (i.e., leaf plus stem mass) at the twig level across very different forest communities (Figure 1A). Additionally, consistent with some previous reports (e.g., Suzuki, 2003), we see that the numerical values of the exponent of*M_L_* vs. *M_T_* and *M_L_* vs. *M_S_* (i.e., 1.01 and 0.99 for twigs) shifts to 0.82 and 0.76 at the level of individual trees (Figure 1B,C). This shift mirrors a shift in the numerical value of the scaling exponent for metabolic rates as the plant body increases in size (i.e., from 1.0 to 3/4; see Niklas, 2004; Reich et al., 2006, Cheng et al., 2010). When these patterns are viewed together, it is reasonable to conclude that metabolic rates likely scale isometrically as a function of current-year twig biomass and will decline as the plant body increases overall, a phenomenon that has been observed before (e.g., Westoby and Wright, 2003; Sun et al., 2006; Cheng and Niklas, 2007; Chave et al., 2014; Paul et al., 2016).

### The Scaling of Leaf Biomass With Respect to Stem Architecture

The data reported here show that leaf mass (*M_L_*) scales approximately as the 2.0-power of stem diameter (*D*), which is similar to the pattern reported by Enquist and Niklas (2002), i.e., total standing leaf mass (*M_L_*) scales as the 1.99-power of basal stem diameter (*D*) at the individual plant level (Table 2 and Figure 2A). In this context, it is useful to note that the ‘Pipe model’ theory argues that branches play a duel role in nutrient transport and mechanical support, such that the number of leaves per twig might be limited by the number of ‘pipe’ units traversing a twig (Shinozaki et al., 1964; Tyree and Ewers, 1991). In this context, Westoby and Wright (2003) argue that the cross-sectional area of twigs is expected to scale with total leaf area for both hydraulic and mechanical reasons. Because some studies report that leaf area scales, on average, isometrically with leaf biomass (West et al., 1997, 1999; Sun et al., 2006), leaf biomass is expected to scale as the 2.0-power of stem diameter at both the individual plant level and the twig level. Moreover, for any equivalent stem diameter, our results show that twigs and individual plants have similar leaf biomass scaling relationships, despite an elevational gradient that affects ambient temperatures (Figure 2A). Although low temperature negatively affect metabolic rates and thus growth (Hoch and Körner, 2012; Mayor et al., 2017) and are expected to modify twig biomass allocation patterns (Xiang et al., 2009a), our results indicate a fixed “invariant” isometric relationship between leaf and stem biomass for twigs regardless of elevation (Figure 1B). Importantly, however, the elevational gradient did influence the numerical value of the normalization constant governing the *M_S_* vs. *D* relationship (Figure 1C). In this context it is important to note that stem bulk tissue density (ρ) differed among the three forests examined in our study, i.e., it was highest in the evergreen forest, which also had the smallest stem diameters per stem biomass (Supplementary Figure 1C).

At the level of an individual plant, the data used in this study reveal a significant correlation between total standing leaf biomass and plant height, but show no clear scaling relationship at the level of individual twigs (Table 2 and Figure 2B), i.e., different relationships between stem length (height) and leaf biomass exist for the twig and whole plant levels (Figure 2C). This finding is consistent with the observation that no single mechanical model exists across plants with and without secondary tissues or over the course of the ontogeny of woody species (Niklas and Spatz, 2004; Enquist et al., 2007; King, 2011). This phenomenology likely reflects the annual amortization of secondary tissues in progressively older stems bearing twigs that typically have little or no secondary tissues.

It is important to cast our results in the context of the hydraulic as well as the mechanical differences between twigs and the architecture of whole trees. First, the height of an individual tree height is not geometrically equivalent to the length of a stem (i.e., twig). The former is influenced by branching angles and the orientation of growth in woody stems as well as twigs, whereas the latter is a simple linear dimension reflecting growth in a single year. Second, water transport through the trunk of a tree is effected by the hydraulic efficiency of sapwood (a secondary tissue), whereas water transport in a twig is governed by the efficiency of primary xylem. Third, the diameter of a tree trunk reflects the accumulation of secondary tissues, which is lacking in twigs, which has many substantive consequences (Ryan and Yoder, 1997; West et al., 1999). Collectively, these and other factors contribute to the fact the scaling of stem diameter (*D*) and stem length (*L*) are fundamentally different at the twig and whole tree levels (Figure 2C).

### The Scaling of Leaf Biomass With Respect to Leaf Number

Our results reveal no significant relationship between leaf number and total leaf mass on twigs (Table 2, *P* > 0.05). According to the leafing intensity (leaf number per unit stem volume) strategy (Kleiman and Aarssen, 2007), average leaf biomass is predicted to decrease with leaf number per stem volume such that individual leaf mass should scale negatively and isometrically with respect to leaf number for given stem volume. This negative scaling relationship might offset the relationship between total twig leaf biomass and leaf number because total twig leaf biomass is the product of leaf number and individual leaf biomass. Thus, leaf number does not invariably correlate with individual leaf mass or individual leaf area (Figure 3B). Furthermore, it is worth noting that our results do not support this prediction that stem length is a primary constraint on twig leaf number (Yagi, 2004) (see Table 3).

## Conclusion

We have demonstrated that isometric scaling relationships hold among leaf biomass, stem biomass, and total twig biomass at the twig level within each of three different forests communities along an elevational gradient. These scaling relationships are insensitive to differences in species composition and variations in environmental conditions along an elevational gradient. Further, consistent with biomass allocation patterns reported for large trees, our analyses show that leaf biomass scales approximately as the 2.0-power of twig stem diameter across the three communities, but fail to reveal a significant relationship between leaf biomass and height/length reported for trees. These observations suggest to us that stem diameter imposes the primary constraint on the overall biomass allocation pattern in twigs because of the nature of hydrodynamic principles governing the transport of essential resources. A growth-hydraulic model rather than a growth-mechanical model appears to govern the scaling relationships among stem length, diameter, and the biomass (Niklas and Spatz, 2004; Fan et al., 2017). Indeed, recent analyses also indicate that tree diameter might be a better predictor of above-ground biomass (Chave et al., 2014; Paul et al., 2016).

## Author Contributions

JS and DC conceived and designed the experiments. JS, MLy, QZ, and MLi performed the experiments. JS and MW analyzed the data. JS, MW, KN, and DC wrote the manuscript. All authors contributed critically to the drafts and gave final approval for publication.

## Conflict of Interest Statement

The authors declare that the research was conducted in the absence of any commercial or financial relationships that could be construed as a potential conflict of interest.

## References

[B1] Blomberg S. P. Garland T. Ives A. (2003). Testing for phylogenetic signal in comparative data: behavioral traits are more labile. *Evolution* 57 717–745. 10.1111/j.0014-3820.2003.tb00285.x 12778543

[B2] Cannell M. G. R. (1982). *World Forest Biomass and Primary Production Data.* London: Academic Press.

[B3] Chave J. Rejou-Mechain M. Burquez A. Chidumayo E. Colgan M. S. Delitti W. B. (2014). Improved allometric models to estimate the aboveground biomass of tropical trees. *Glob. Chang. Biol.* 20 3177–3190. 10.1111/gcb.12629 24817483

[B4] Cheng D. L. Li T. Zhong Q. L. Wang G. X. (2010). Scaling relationship between tree respiration rates and biomass. *Biol. Lett.* 6 715–717. 10.1098/rsbl.2010.0070 20356882 PMC2936133

[B5] Cheng D. L. Niklas K. J. (2007). Above- and below-ground biomass relationships across 1534 forested communities. *Ann. Bot.* 99 95–102. 10.1093/aob/mcl206 17085476 PMC2802968

[B6] Enquist B. J. Allen A. P. Brown J. H. Gillooly J. F. Kerkhoff A. J. Niklas K. J. (2007). Biological scaling: does the exception prove the rule? *Nature* 445 E9–E10. 17268426 10.1038/nature05548

[B7] Enquist B. J. Niklas K. J. (2002). Global allocation rules for patterns of biomass partitioning in seed plants. *Science* 295 1517–1520. 10.1126/science.1066360 11859193

[B8] Fan Z. X. Sterck F. Zhang S. B. Fu P. L. Hao G. Y. (2017). Tradeoff between stem hydraulic efficiency and mechanical strength affects leaf-stem allometry in 28 *Ficus* tree species. *Front. Plant Sci.* 8:1619. 10.3389/fpls.2017.01619 28979282 PMC5611361

[B9] Grace J. B. Michael A. T. Smith M. D. Seabloom E. Andelman S. J. Meche G. (2007). Does species diversity limit productivity in natural grassland communities? *Ecol. Lett.* 10 680–689. 17594423 10.1111/j.1461-0248.2007.01058.x

[B10] Hoch G. Körner C. (2012). Global patterns of mobile carbon stores in trees at the high-elevation tree line. *Glob. Ecol. Biogeogr.* 21 861–871. 10.1111/j.1466-8238.2011.00731.x

[B11] Huang Y. Lechowicz M. J. Price C. A. Li L. Wang Y. Zhou D. (2015). The underlying basis for the trade-off between leaf size and leafing intensity. *Funct. Ecol.* 30 199–205. 10.1111/1365-2435.12491

[B12] Kembel S. W. Cowan P. D. Helmus M. R. Cornwell W. K. Morlon H. Ackerly D. D. (2010). Picante: R tools for integrating phylogenies and ecology. *Bioinformatics* 26 1463–1464. 10.1093/bioinformatics/btq166 20395285

[B13] King D. A. (2011). “Size-related changes in tree proportions and their potential influence on the course of height growth,” in *Size- and Age-Related Changes in Tree Structure and Function*, eds Meinzer F. C. Lachenbruch B. Dawson T. E. (Dordrecht: Springer), 165–191. 10.1007/978-94-007-1242-3_6

[B14] Kleiman D. Aarssen L. W. (2007). The leaf size/number trade-off in trees. *J. Ecol.* 95 376–382. 10.1111/j.1365-2745.2006.01205.x

[B15] Li M. Zheng Y. Fan R. R. Zhong Q. L. Cheng D. L. (2017). Scaling relationships of twig biomass allocation in *Pinus hwangshanensis* along an altitudinal gradient. *PLoS One* 12:e0178344. 10.1371/journal.pone.0178344 28552954 PMC5446166

[B16] Mayor J. R. Sanders N. J. Classen A. T. Bardgett R. D. Clement J. C. Fajardo A. (2017). Elevation alters ecosystem properties across temperate treelines globally. *Nature* 542 91–98. 10.1038/nature21027 28117440

[B17] Milla R. (2009). The leafing intensity premium hypothesis tested across clades, growth forms and altitudes. *J. Ecol.* 97 972–983. 10.1111/j.1365-2745.2009.01524.x

[B18] Niklas K. J. (1992a). Gravity-induced effects on material properties and size of leaves on horizontal shoots of acer saccharum (aceraceae). *Am. J. Bot.* 79 820–827. 10.1002/j.1537-2197.1992.tb13659.x

[B19] Niklas K. J. (1992b). *Plant Biomechanics: An Engineering Approach to Plant form and Function.* Chicago, IL: University of Chicago press.

[B20] Niklas K. J. (2004). Plant allometry: is there a grand unifying theory? *Biol. Rev.* 79 871–889. 10.1017/S1464793104006499 15682874

[B21] Niklas K. J. Enquist B. J. (2002a). Canonical rules for plant organ biomass partitioning and annual allocation. *Am. J. Bot.* 89 812–819. 10.3732/ajb.89.5.812 21665681

[B22] Niklas K. J. Enquist B. J. (2002b). On the vegetative biomass partitioning of seed plant leaves, stems, and roots. *Am. Nat.* 159 482–497. 10.1086/339459 18707431

[B23] Niklas K. J. Spatz H. C. (2004). Growth and hydraulic (not mechanical) constraints govern the scaling of tree height and mass. *Proc. Natl. Acad. Sci. U.S.A.* 101 15661–15663. 10.1073/pnas.0405857101 15505224 PMC524850

[B24] Niklas K. J. Spatz H. C. (2010). Worldwide correlations of mechanical properties and green wood density. *Am. J. Bot.* 97 1587–1594. 10.3732/ajb.1000150 21616793

[B25] Olson M. E. Aguirrehernández R. Rosell J. A. (2009). Universal foliage-stem scaling across environments and species in dicot trees: plasticity, biomechanics and Corner’s rules. *Ecol. Lett.* 12 210–219. 10.1111/j.1461-0248.2008.01275.x 19141123

[B26] Paradis E. Claude J. Strimmer K. (2004). Ape: analyses of phylogenetics and evolution in R language. *Bioinformatics* 20 289–290. 10.1093/bioinformatics/btg412 14734327

[B27] Paul K. I. Roxburgh S. H. Chave J. England J. R. Zerihun A. Specht A. (2016). Testing the generality of above-ground biomass allometry across plant functional types at the continent scale. *Glob. Chang. Biol.* 22 2106–2124. 10.1111/gcb.13201 26683241

[B28] Pickup M. Westoby M. Basden A. (2005). Dry mass costs of deploying leaf area in relation to leaf size. *Funct. Ecol.* 19 88–97. 10.1111/j.0269-8463.2005.00927.x

[B29] R Core Team (2012). *R: A Language and Environment for Statistical Computing.* Vienna: R Foundation for Statistical Computing.

[B30] Reich P. B. Tjoelker M. G. Machado J. L. Oleksyn J. (2006). Universal scaling of respiratory metabolism, size and nitrogen in plants. *Nature* 439 457–461. 10.1038/nature04282 16437113

[B31] Ryan M. G. Yoder B. J. (1997). Hydraulic limits to tree height and tree growth. *BioScience* 47 235–242. 10.2307/1313077

[B32] Shinozaki K. Yoda K. Hozumi K. Kira T. (1964). A quantitative analysis of plant form: the pipe model theory. II. Further evidence of the theory and its application in forest ecology. *Jpn. J. Ecol.* 14 133–139.

[B33] Smith D. D. Sperry J. S. Adler F. R. (2017). Convergence in leaf size versus twig leaf area scaling: do plants optimize leaf area partitioning? *Ann. Bot.* 119 447–456. 10.1093/aob/mcw231 28028019 PMC7296615

[B34] Sun S. Jin D. Shi P. (2006). The leaf size-twig size spectrum of temperate woody species along an altitudinal gradient: an invariant allometric scaling relationship. *Ann. Bot.* 97 97–107. 10.1093/aob/mcj004 16254019 PMC2803375

[B35] Suzuki M. (2003). Size structure of current-year shoots in mature crowns. *Ann. Bot.* 92 339–347. 10.1093/aob/mcg144 12853280 PMC4257506

[B36] Tyree M. T. Ewers F. W. (1991). The hydraulic architecture of trees and other woody-plants. *New Phytol.* 119 345–360. 10.1111/j.1469-8137.1991.tb00035.x

[B37] Warton D. I. Duursma R. A. Falster D. S. Taskinen S. (2012). Smatr 3-an R package for estimation and inference about allometric lines. *Methods Ecol. Evol.* 3 257–259. 10.1111/j.2041-210X.2011.00153.x

[B38] Warton D. I. Wright I. J. Falster D. S. Westoby M. (2006). A review of bivariate line-fitting methods for allometry. *Biol. Rev.* 81 259–291. 10.1017/S1464793106007007 16573844

[B39] West G. B. Brown J. H. Enquist B. J. (1997). A general model for the origin of allometric scaling laws in biology. *Science* 276 122–126. 10.1126/science.276.5309.122 9082983

[B40] West G. B. Brown J. H. Enquist B. J. (1999). The fourth dimension of life: fractal geometry and allometric scaling of organisms. *Science* 284 1677–1679. 10.1126/science.284.5420.1677 10356399

[B41] Westoby M. Falster D. S. Moles A. T. Vesk P. A. Wright I. J. (2002). Plant ecological strategies: some leading dimensions of variation between species. *Ann. Rev. Ecol. Syst.* 33 125–159. 10.1146/annurev.ecolsys.33.010802.150452

[B42] Westoby M. Wright I. J. (2003). The leaf size-twig size spectrum and its relationship to other important spectra of variation among species. *Oecologia* 135 621–628. 10.1007/s00442-003-1231-6 16228258

[B43] Wright I. J. Falster D. S. Pickup M. Westoby M. (2006). Cross-species patterns in the coordination between leaf and stem traits, and their implications for plant hydraulics. *Physiol. Plant.* 127 445–456. 10.1111/j.1399-3054.2006.00699.x

[B44] Xiang S. Liu Y. L. (2009b). Stem architectural effect on leaf size, leaf number, and leaf mass fraction in plant twigs of woody species. *Int. J. Plant Sci.* 170 999–1008. 10.1086/605114

[B45] Xiang S. Wu N. Sun S. C. (2009a). Within-twig biomass allocation in subtropical evergreen broad-leaved species along an altitudinal gradient: allometric scaling analysis. *Trees* 23 637–647. 10.1007/s00468-008-0308-6

[B46] Yagi T. (2000). Morphology and biomass allocation of current-year shoots of ten tall tree species in cool temperate Japan. *J. Plant Res.* 113 171–183. 10.1007/PL00013928

[B47] Yagi T. (2004). Within-tree variations in shoot differentiation patterns of 10 tall tree species in a Japanese cool-temperate forest. *Can. J. Bot.* 82 228–243. 10.1139/b03-124

[B48] Yang Y. He X. Xu X. Yang D. (2015). Scaling relationships among twig components are affected by sex in the dioecious tree *Populus cathayana*. *Trees* 29 737–746. 10.1007/s00468-014-1151-6

[B49] Zhang Z. Zhong Q. Niklas K. J. Liang C. Yang Y. Cheng D. (2016). A predictive nondestructive model for the covariation of tree height, diameter, and stem volume scaling relationships. *Sci. Rep.* 6:31008. 10.1038/srep31008 27553773 PMC4995560

