# Supplementary material for: Stem Diameter (and Not Length) Limits Twig Leaf Biomass
Source: Front Plant Sci. 2019 Feb 21;10:185. doi: 10.3389/fpls.2019.00185 (PMC6393343; doi:10.3389/fpls.2019.00185)

Supplementary

Table S1 Phylogenetic signal of twig functional traits test in three forests.

Functional traits	*K*	*P*
Total twig mass (M_T)	0.042	0.684
Total leaf mass (M_L)	0.042	0.678
Stem mass (M_S)	0.044	0.657
Stem diameter (D)	0.09	0.479
Stem length (L)	0.066	0.309
Stem volume (V)	0.057	0.585
Leaf number (N_L)	2.488	0.001

The detailed description of *K* statistics see Blomberg et al. (2003).

Figure 1. Bivariate plots among the leaf, stem, and total mass at twig level. A: the relationships among the leaf, stem and total mass; B: the relationship between leaf and stem mass; C: the relationship between stem mass and stem diameter; D: the relationship between stem mass and stem length.

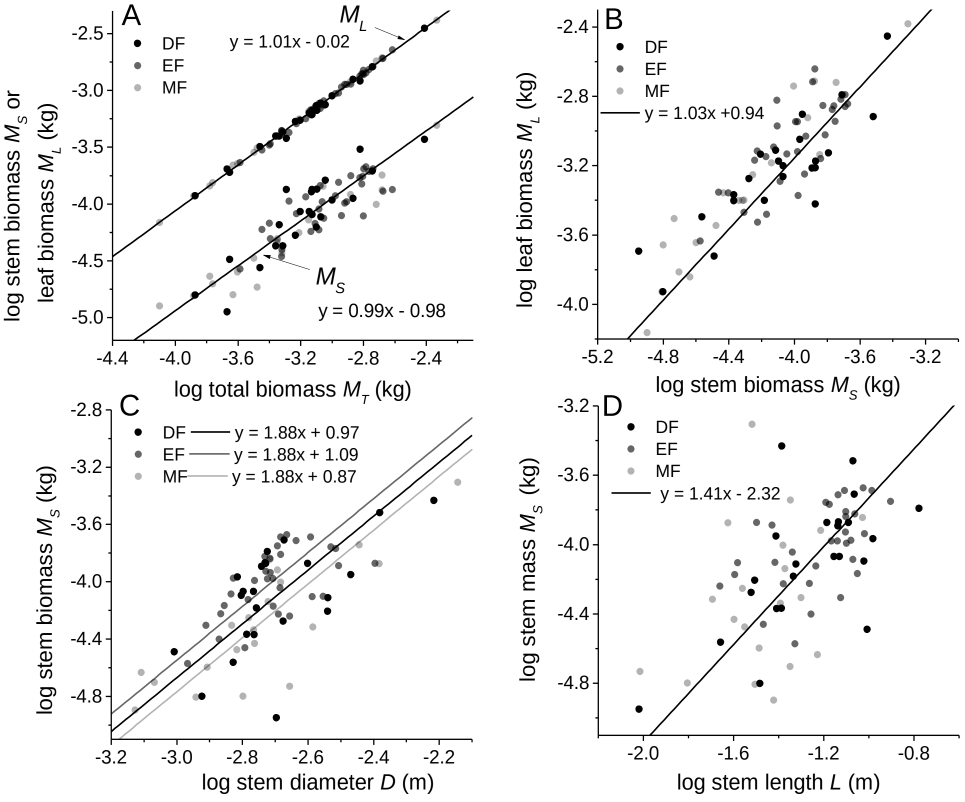

Figure 2. Bivariate plot of leaf biomass versus stem diameter at the twig level of three forests.

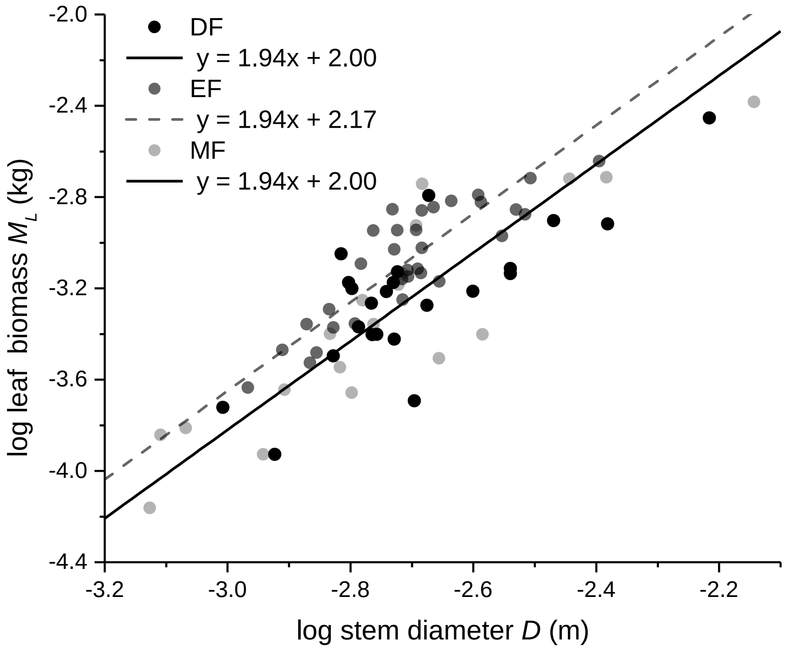

Figure 3. Bivariate plot of leaf biomass versus leaf number at the twig level of three forests. "NS" denotes a statistically non-significant relationship.

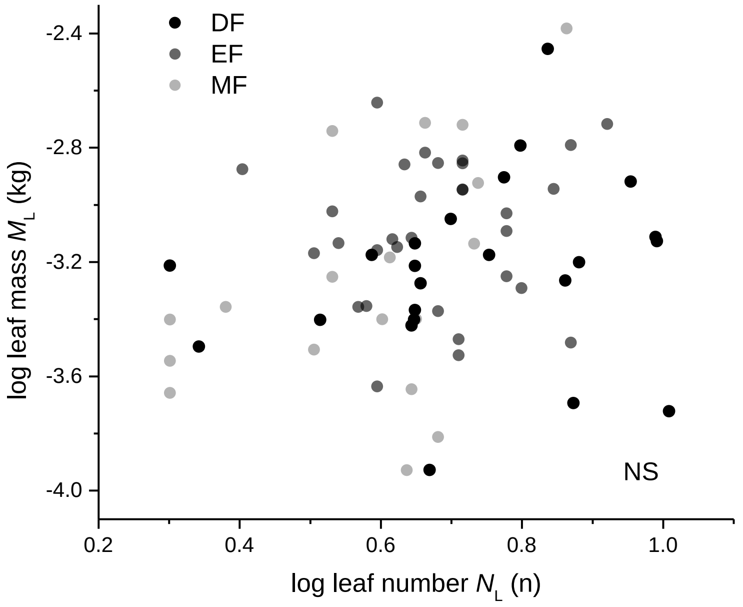

Figure 4. Correlation of evolutionary divergences with leaf number and other twig traits.

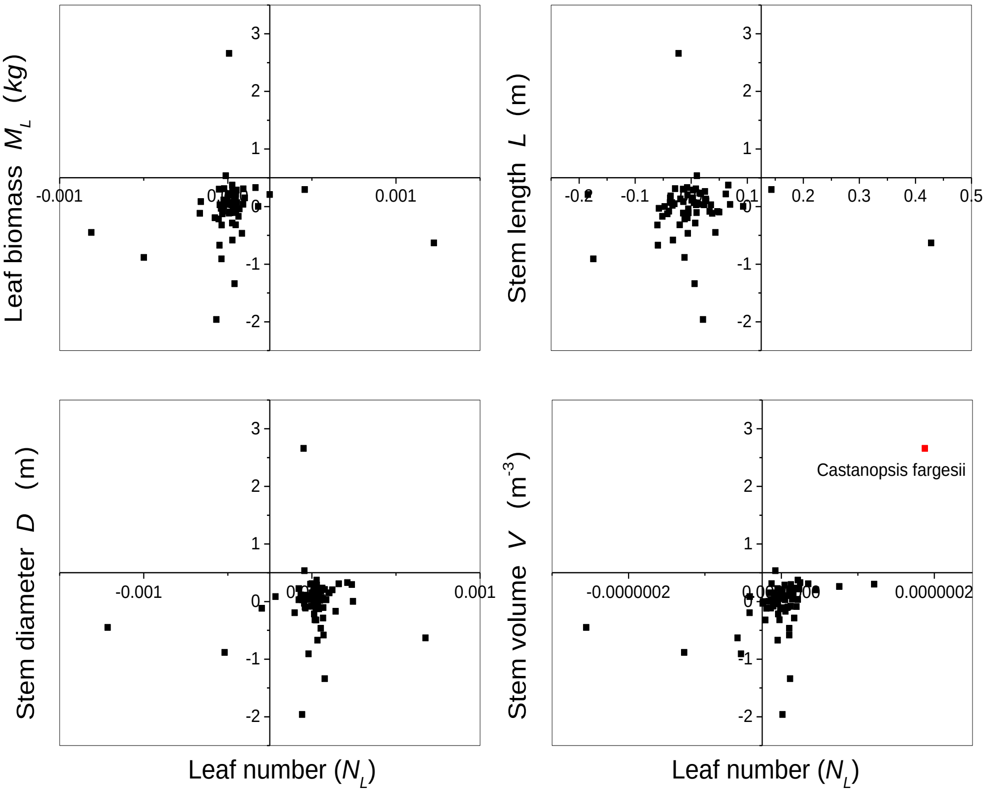

Supplement: Supplementary file 1 [file Data_Sheet_1.PDF]
